# Subcutaneous Rupture of the Extensor Pollicis Longus Tendon after Corticosteroid Injections for DeQuervain's Stenosing Tenovaginitis

**DOI:** 10.1155/2014/934384

**Published:** 2014-10-12

**Authors:** Hassan Boussakri, Amara Bouali

**Affiliations:** ^1^Montpellier Institute of Surgery for the Hand, Clinical Clementville, 34000 Montpellier, France; ^2^Department of Orthopaedic Surgery (B4), CHU Hassan II Hospital, University of Sidi Mohammed Ben Abdellah, 3000 Fez, Morocco

## Abstract

DeQuervain's stenosing tenovaginitis is a common condition. Nonsurgical treatment by corticosteroid injection has significantly improved the management of this disease. The authors describe a case of subcutaneous rupture of the extensor pollicis longus tendon at the wrist, three months after two corticosteroid injections for DeQuervain's stenosing tenovaginitis. The etiological history has not found any trauma history of the wrist. The aim of our work is to draw attention to this rare complication and discuss its therapeutic management. Our functional results were excellent.

## 1. Introduction

The DeQuervain's stenosing tenovaginitis (STV) is a pathology caused by repetitive overuse of the abductor pollicis longus and extensor pollicis brevis tendons on the inside of the first dorsal compartment of the wrist. Nonsurgical treatment by corticosteroid injection significantly improved the management of this disease. Hand surgeons should be aware of the possibility of tendon rupture either in the first extensor compartment or remotely at other compartments in proximity.

Spontaneous rupture of the extensor pollicis longus tendon at wrist level is rare and is mostly reported after distal radius fracture at the Lister tubercle level or after a medical condition such as rheumatoid arthritis [1]. But there are few publications concerning the rupture of the extensor pollicis longus tendon at the wrist, after local injection of corticosteroids [2, 3]. This rupture of the tendon at the injection site usually occurs one or two weeks after the corticosteroid injection [4].

The authors describe a case of rupture of the extensor pollicis longus tendon, three months after two corticosteroid injections for DeQuervain's stenosing tenovaginitis (STV), and the etiological history did not find a previous trauma. The aim of our work is to draw attention to this rare complication and discuss its therapeutic management.

## 2. Observation

This is a young woman, aged 26, left-handed, who works as a nurse, with no special medical or traumatic history. She was addressed to specialized hand surgery consultation for a complete inability to extend the thumb of the right hand, which occurred three months after the corticosteroid injection. The patient experienced a crackling sensation associated with brutal pain localized at the metacarpal phalangeal level while hanging an infusion. This symptom was followed by complete inability to extend the thumb. The patient reported no history of trauma or other risk factors that could explain the rupture of the extensor pollicis longus tendon except the corticosteroid injection. Based on clinical interview data, the patient reported that, in those three months before the event, she had been treated for DeQuervain's stenosing tenovaginitis (STV) receiving two corticosteroid injections 20 days apart, with immobilisation performed by her doctor.

**Figure 1:**
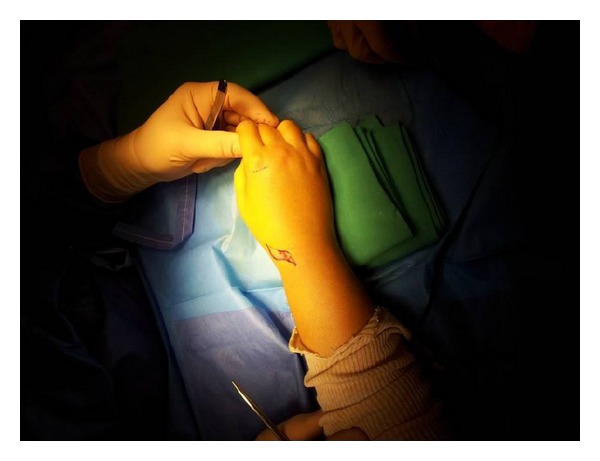
Clinical examination shows a thumb in flexion position.

Clinical examination of the hand and the wrist found a thumb in flexion position (Figure 1); the patient was unable to actively extend the thumb in the metacarpophalangeal joint and the distal phalanx. Normally during passive hyperextension, the extensor pollicis longus tendon is visible and palpable subcutaneously, while in our case the tendon is neither palpable nor visualized.

**Figure 2:**
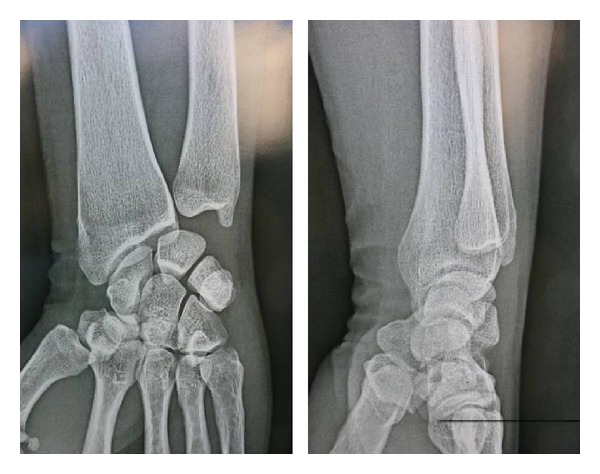
X-ray of the hand shows no bony abnormality.

The examination of the contralateral hand was normal. The X-ray of the hand showed no bony abnormality (Figure 2) and the ultrasonography confirmed tendon rupture at the level of the anatomical snuff box.

**Figure 3:**
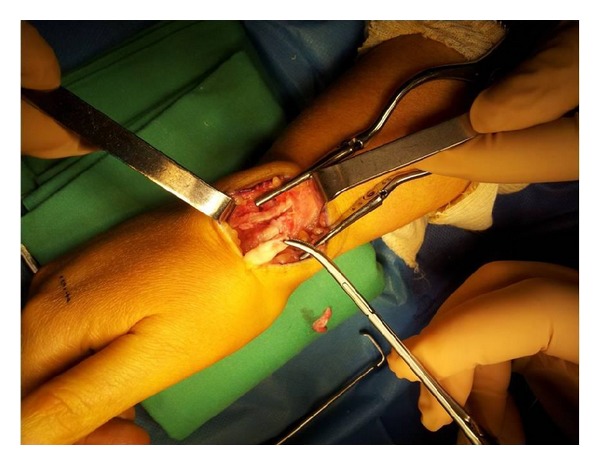
Intraoperative view showing a total rupture of the extensor pollicis longus tendon with a 10% diameter reduction of the extensor carpi radialis longus tendon without rupture.

**Figure 4:**
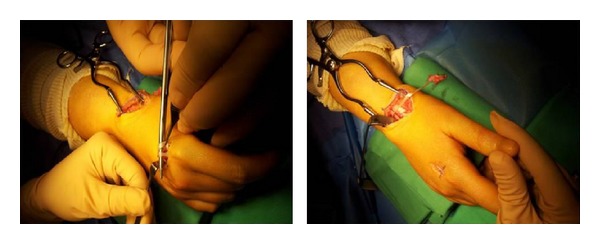
Taking of the extensor indicis proprius tendon by a second incision on the dorsal surface of the metacarpophalangeal joint of the index finger.

**Figure 5:**
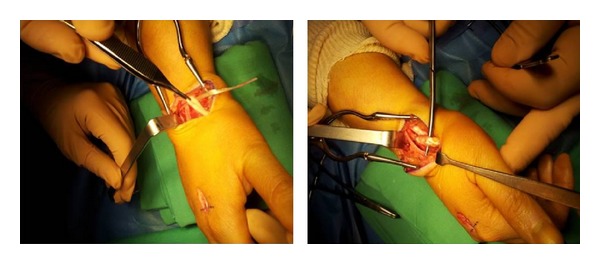
Intraoperative view after final adjustment and reconstruction of the extensor pollicis longus tendon.

*Surgical Technique*. Surgery is decided and performed under local anesthesia, by axillary brachial plexus block, a pneumatic tourniquet being inflated and placed at the limb radix. Surgical approach was focused on the anatomical snuff box. Intraoperatory view, we found a total rupture of the extensor pollicis longus tendon, the edges of this tendon were visualized but frayed, and there was a retraction of the proximal end. We also found about 10% diameter reduction of the extensor carpi radialis longus tendon without tendon rupture (Figure 3). We note the absence of the macroscopic sign of synovitis or tenosynovitis (Figure 3). The edges of the extensor pollicis longus tendon were debrided. The reconstruction of the tendon was performed using the extensor indicis proprius tendon, by a second incision on the dorsal surface of the metacarpophalangeal joint of the index finger (Figure 4). The distal end of the extensor indicis proprius tendon was sutured to the extensor digitorum communis, but the proximal stump was sutured to the distal end of the extensor pollicis longus, using a 3-0 nylon thread and then by continuous suture with 5-0 nylon monofilament thread, according to Pulvertaft repair technique (Figure 5).

The adjustment was set in the position: 0° extension in the metacarpophalangeal and interphalangeal joints of the thumb, with the wrist in neutral position (Figure 5). The tension of the repair was considered satisfactory when the extension of the thumb occurred with retraction of the reconstructed tendon under the effect of tenodesis during wrist flexion and when full flexion of the thumb with wrist extension was possible. Immobilization was performed by applying a circular plaster on the forearm, with the wrist in neutral position and the thumb being abducted and extended for a period of 4 weeks, and then rehabilitation by physical therapy with gentle movements and protection by intermittent splint. At 6 weeks postoperative period, the splint was removed completely. In the last follow-up at 3 months, the patient was able to complete the extension of the metacarpophalangeal and interphalangeal joint of the thumb. The quick DASH score was 42 points before surgery and 15 after the surgery.

## 3. Discussion

The DeQuervain's stenosing tenovaginitis (STV) is a pathology caused by repetitive overuse of the abductor pollicis longus and extensor pollicis brevis tendons on the inside of the first dorsal compartment of the wrist. Nonsurgical treatment for the STV brought satisfactory results; corticosteroids injections alone have a success rate between 68% and 100% and a significant clinical improvement compared to other treatment modalities such as the use of non-steroidal anti-inflammatory drugs, with a success rate of 0% [5]. The use of corticosteroid injections is the most effective nonsurgical treatment for STV, with a low rate of adverse effects, but which can be severe. Hand surgeons should be aware of the possibility of tendon rupture either in the first extensor compartment or remotely at other compartments at proximity. The first case of subcutaneous rupture of the extensor pollicis longus was described in 1876 by Duplay, associated with Pouteau-Colles fracture [6], whose pathophysiological explanation is purely mechanical. After this publication, several etiologies have been described: tendon rupture in rheumatoid arthritis [1], scaphoid fracture, scaphoid pseudoarthrosis and associated with the subluxation of the distal radioulnar joint [2, 7] and same cases with particular professional activities without predisposing factors [8]. However, rare cases have been reported on tendon rupture after corticosteroid injection [4, 9], because it is difficult to make an etiological diagnosis for the association between inflammatory disease and the use of corticosteroids [2]. No similar cases or documents were previously reported or published in the literature about spontaneous rupture of the extensor pollicis longus tendon after corticosteroid injection for the DeQuervain's stenosing tenovaginitis. The subcutaneous rupture of the extensor pollicis longus tendon usually occurs one or two weeks after corticosteroid injection in the first extensor compartment [5]. However, the rupture of the extensor pollicis longus tendon has been reported in association with several local corticosteroid injections [9]. In our case, the patient received two corticosteroid injections 3 months previously, the dose not being specified, on the posterior side of the wrist causing a spontaneous rupture of the extensor pollicis longus. We believe that the rupture of the extensor pollicis longus tendon at the 3rd extensor compartment of the wrist after steroid injections for STV was explained by the anatomical proximity of the two compartments (1st and 3rd compartments) at the dorsal side of the wrist and the dissemination and long elimination of the corticosteroids in the soft tissue. This rupture spontaneous of the tendon was followed by a complete inability to extend the thumb, and the history has not objectified any trauma history to the wrist, which may explain this complication. In the limit of our bibliographic research, there are rare publications reviewing the tendon complications of the hand after local corticosteroid injections [10]. Fredberg reported that when corticosteroids are administered within the tendon tissue, they can have adverse effects on tendon fibers causing tendon degeneration by altering collagen synthesis [11]. Although the injection of corticosteroids is relatively safe and is widely used with success to treat the STV, they should be administered with great caution [12].

## 4. Conclusion

In conclusion, we are reminded that corticosteroid injections for the treatment of DeQuervain's stenosing tenovaginitis are relatively easy to perform, but mismanagement could lead to serious consequences such as spontaneous rupture of the extensor pollicis tendon rupture, which may require surgical repair followed by complete inability to use the hand with a long duration of immobilization and rehabilitation.

## Abbreviations

STV: DeQuervain's stenosing tenovaginitis.
